# The Evolution of Intein-Based Affinity Methods as Reflected in 30 years of Patent History

**DOI:** 10.3389/fmolb.2022.857566

**Published:** 2022-04-08

**Authors:** Sai Vivek Prabhala, Izabela Gierach, David W. Wood

**Affiliations:** ^1^ William G. Lowrie Department of Chemical and Biomolecular Engineering, The Ohio State University, Columbus, OH, United States; ^2^ Protein Capture Science, Columbus, OH, United States

**Keywords:** inteins, protein purification, patents, split inteins, protein engineering

## Abstract

Self-cleaving affinity tags, based on engineered intein protein domains, have been touted as a universal single step purification platform for tagless non-mAb proteins. These approaches provide all of the power and flexibility of tag-based affinity methods, but deliver a tagless target protein suitable for clinical applications without complex process development. This combination of features might accelerate and de-risk biopharmaceutical development by bridging early discovery to full-scale manufacturing under a single platform. Despite this profound promise, intein-based technologies have yet to reach their full potential. This review examines the evolution of intein-based purification methods in the light of several significant intein patents filed over the last 3 decades. Illustrated with actual key figures from each of the relevant patents, key advances are described with a focus on applications in basic research and biopharmaceutical production. Suggestions for extending intein-based purification systems to emerging therapies and non-protein applications are presented as concluding remarks.

## A Brief Introduction to Inteins

Inteins (**In**tervening pro**teins**) are a family of protein segments found in nature that are capable of posttranslational splicing. They were first reported in 1990 (Hirata et al., 1990; Kane et al., 1990), and were soon shown to have the ability to spontaneously excise themselves from larger polypeptide precursors through a process analogous to intron self-splicing. This reaction releases the intein from the precursor protein while ligating the flanking sequences (referred to as exteins) by a native peptide bond. Importantly, the intein splicing reaction is self-contained and many inteins exhibit robust splicing outside of their native genetic context and in the absence of any cofactors. Because of these capabilities, inteins were quickly recognized as potentially valuable tools for protein engineering and purification, and early intein patents focused primarily on these applications.

**FIGURE 1:**
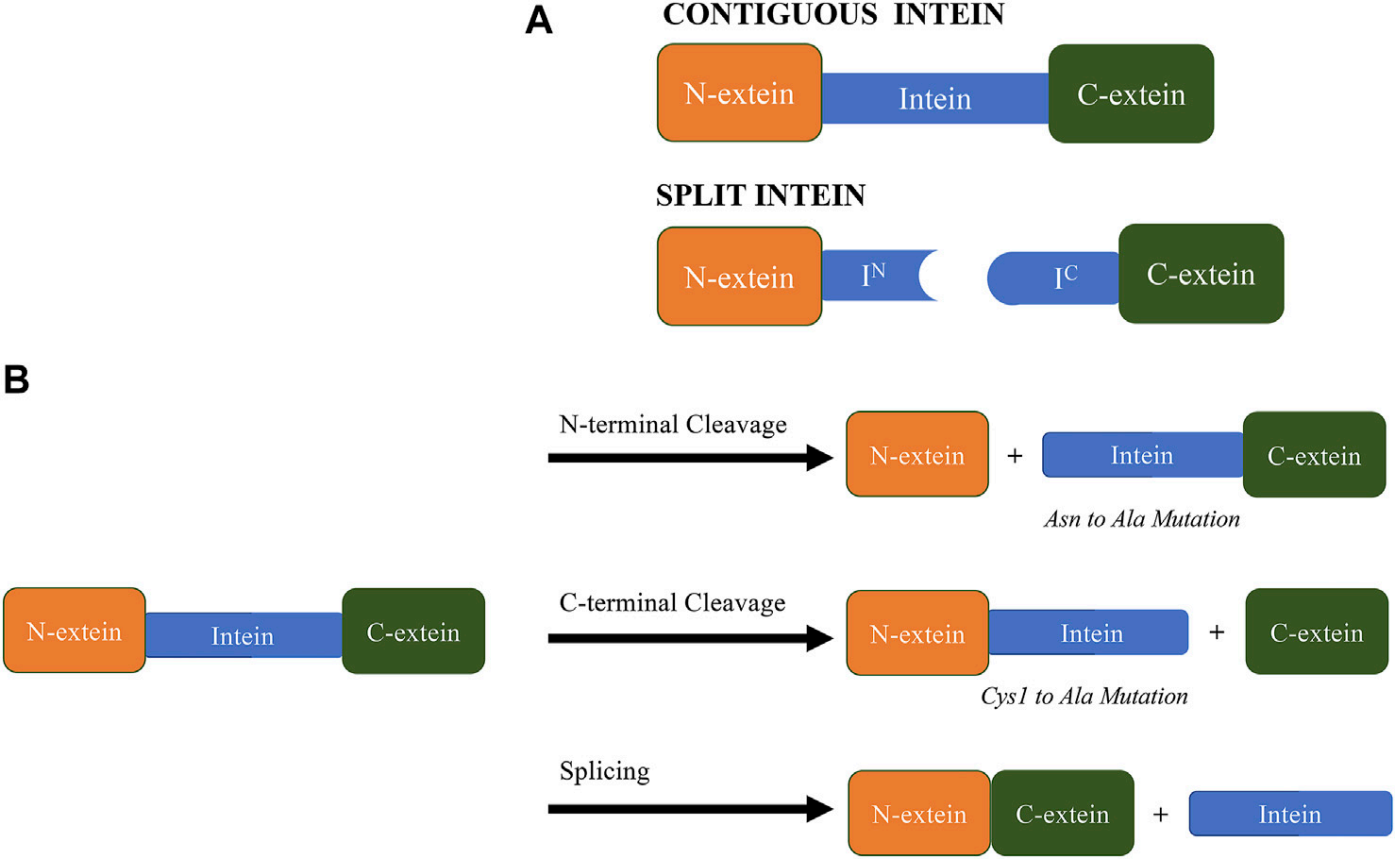
**(A)**. The drawing of split and contiguous inteins. **(B)**. Examples of products obtained from different intein splicing and cleaving reactions. It is important to note that mutation of the last asparagine and the first cysteine (or Ser1) to alanine render most inteins N- and C-terminal cleaving. Other critical residues within catalytic center affecting activity of inteins, including His429/His125, His73/His72, Thr70/69 and Asp422/Asp118 from *Mycobacterium tuberculosis* RecA (PDB ID 2IMZ) and *Nostoc punctiforme* (PDB ID 2KEQ), respectively (Van Roey et al., 2007; Oeemig et al., 2009; Frutos et al., 2010; Ramirez et al., 2013; Eryilmaz et al., 2014; Wood & Camarero, 2014; Cronin et al., 2015).

Inteins are broadly classified into two categories: contiguous inteins and split inteins (Figure 1A). Contiguous inteins result from the transcription and translation of a single gene and constitute the vast majority of all known inteins. On the other hand, split inteins are transcribed from two different genes in a single cell, resulting in two different transcripts that are separately translated as the N-extein-N-intein fragment and the C-intein-C-extein fragment. The intein fragments then spontaneously assemble (non-covalently) into an active splicing complex, resulting in the spliced product and the excised split inteins (F. B. Perler, 2002). Split inteins can be naturally-occurring or artificial, where in the latter case a contiguous intein segment is usually split at an optimal location for a specific application.

Bioinformatics approaches have identified inteins embedded in critical coding regions within genomes across Archaea, Bacteria and Eukarya. For a long time, the dominant theory for explaining the existence of inteins has been that they are selfish genetic elements that mediate their own spread across various genomes through their embedded homing endonuclease domains (Derbyshire et al., 1997). Another theory states that nature evolved these self-splicing units for regulated assembly of important proteins as they are found embedded in sequences that are responsible for DNA replication, transcription and maintenance, as well as in other key metabolic enzymes (Pietrokovski, 2001). Interestingly, many intein sequences are located within highly conserved sites that are critical to the function of the host protein, such as the active sites of enzymes. Some inteins have also been implicated in host protein function and regulation (Green et al., 2019). In most native contexts, the splicing reaction is spontaneous and does not require any external stimulus. However, in some inteins it has been shown that *in vivo* protein splicing can be affected by a wide range of cellular stressors, inhibitors, stimulators and changes in environmental conditions such as pH, osmolarity and temperature (Nicastri et al., 2013; Belfort, 2017; Lennon et al., 2021). Among the recognized protein splicing modulators are divalent cations, salts, exposure to reactive oxygen and nitrogen species and the presence of other small molecules such as flavins. It is also known that protein splicing can be triggered as a response to DNA and ssDNA damage, and in some cases can be regulated by photoactivation and change of redox state (Topilina and Mills, 2014; Lennon et al., 2016).

In addition to being categorized as contiguous or split, inteins are also distinguished based on differences in their conserved active-site residues and resulting variations of the canonical splicing mechanism. The three major classes of inteins are Class 1, Class 2, and Class 3, where Class 1 inteins are the most common and have been studied the most extensively (Mills et al., 2014; Tori and Perler, 2017). The most widely accepted splicing mechanism for Class 1 inteins consists of four sequential nucleophilic attacks that are mediated by highly conserved intein and extein residues at the splice junctions (Perler, 2002). During step 1, an acyl-shift at the N-terminal intein-extein junction results in cleavage of the upstream peptide bond to form an ester or thioester bond between the N-extein and intein (depending on the sidechain of the first intein residue: Cys, Ser or Thr). In step 2, the hydroxyl (-OH) or thiol (-SH) functional group of the first C-extein residue (a conserved Cys, Thr or Ser) attacks the thioester bond formed in step 1, resulting in a transesterification reaction where the N-extein is transferred to the side chain of the first C-extein residue. This effectively ligates the two exteins with an ester bond. In step 3, the C-terminal intein residue (typically a conserved Asn), releases the linked exteins via formation of a succinimide ring that cleaves the downstream peptide bond. In the final step 4, the thioester bond linking the two exteins spontaneously rearranges to a peptide bond by a O-N or S-N acyl shift to form the spliced product (Mills et al., 2014).

Based on the mechanism above, we can understand how the N-terminal cleavage reaction (via acyl shift) and C-terminal cleavage reaction (via succinimide formation) are distinct, and mutations at the N- and C-terminal intein-extein junctions have been used to promote side-reactions that include cleavage at either or both junctions without extein ligation (Figure 1B) (Amitai et al., 2009). Intein mutants that exhibit isolated and controlled cleaving reactions were initially combined with conventional affinity tags to render the tags self-cleaving. Subsequent split intein systems have retained the ability to exhibit isolated and controlled cleaving but provide additional control of cleaving via intein segment assembly. These approaches have been used to develop a number of intein-based purification systems and are the subject of several intein patents over the past 25 years.

This review focuses on how inteins came to be used for protein purification, the modifications made to early intein designs, and the evolution of new inteins for industrial use as described in prominent intein patents filed over the last 3 decades. To better illustrate the remarkable evolution of intein technology, figures adapted from key patents are included to highlight major advances. Many additional reviews on intein biology and applications are available to describe the fundamental research that led to these patents (Topilina and Mills, 2014; Wood and Camarero, 2014; Pavankumar, 2018).

## Affinity Methods Are Very Efficient but Major Issues Have Prevented Scale up

Techniques such as lectin affinity chromatography (LAC) and immunoaffinity chromatography (IAC) are widely used at laboratory scale for the purification of various proteins. LAC makes use of immobilized lectins which have high affinity for glycan moieties decorating glycoprotein surfaces (O’Connor et al., 2017). On the other hand, IAC makes use of strong, non-covalent interactions between monoclonal/polyclonal antibodies and their antigens (Moser and Hage, 2010). Protein A affinity methods provide a major platform for the purification of monoclonal antibodies in the biopharmaceutical industry, where an immobilized Protein A ligand on a resin backbone selectively binds to the highly conserved Fc antibody domain. While these techniques are very selective in nature and involve resins that can bind strongly to a protein of interest (POI), they can only be applied for certain categories of biologics. Furthermore, production of these resins is capital intensive which has limited their use to laboratory scale.

Many proteins have no conserved features than lend themselves to a native affinity platform, but these platforms can still be introduced through the genetic fusion of an affinity tag to the target protein before expression. In this case a fusion protein is expressed, where the affinity tag can be selectively captured via a corresponding affinity resin, allowing the fused target protein to also be purified. Although many different tags have been developed over the past several decades, the most commonly used at research scale involves strong and specific interactions between polyhistidine sequences and chelated metals. In this approach, known as immobilized metal affinity chromatography (IMAC), a fused affinity tag made up a of series of histidine residues on the POI is selectively captured by an immobilized metal ion on the IMAC resin. Once the soluble impurities are washed away, the POI and fused tag can be eluted from the resin at high purity and yield, which is typically achieved using either a change in pH or by addition of imidazole, which displaces the tag from the resin (Cheung et al., 2012).

Although affinity tag methods have become highly popular in laboratories throughout the world, where dozens of tags and corresponding resins are commercially available, they possess significant drawbacks for clinical applications at large scale. Primary among these is that once the POI has been purified, an additional step is required when removal of the affinity tag is desired. Tag removal is particularly critical for proteins destined for clinical applications due to their potential immunogenicity in the human body. Most often, affinity tag removal is achieved either by using non-specific exopeptidases like carboxypeptidase, or by more highly specific serine proteases like factor Xa and enterokinase. More recent proteases like WELQut and SUMO can cleave a target without leaving additional amino acids as long as they are located at the appropriate terminus of the target POI. Frequently, the protease reaction must be optimized to balance unwanted cleavage within the target protein and the desired separation of the fusion partner. Another associated problem is the need for an additional purification step to isolate the POI from the cleaved affinity tag and the protease used, although this has also been largely solved via the strategic addition of His tags on commercially available protease enzymes. Despite these advances however, the prohibitive cost of highly selective protease enzymes and the long reaction times required for cleavage are two major limitations that continue to prevent economic scale up of affinity tag-based methods for the purification of native POIs. Thus, solving the tag removal problem could enable cheap, scalable, and universal affinity tag methods for research and large-scale biologics production.

## Modified Inteins Have Been Proposed as a Viable Platform for Tag Removal

**FIGURE 2:**
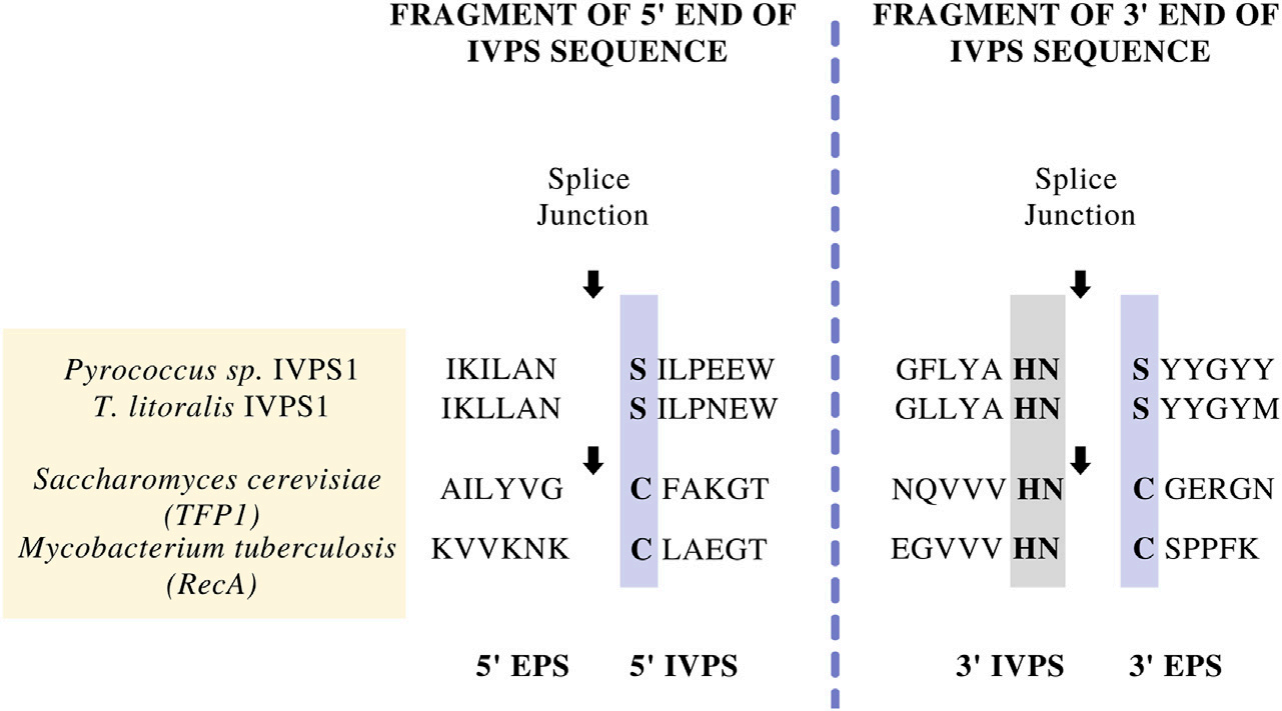
Examples of the amino acid sequences at the protein splice junctions that were used to initially define intein segments by New England Biolabs team (Comb et al., 1996; Patent No.: US 5496714 A; filed in 1992, approved in 1996 and expired in 2013). In the splicing mechanism, the N-terminal and C-terminal splice junctions have a common set of amino acids that are conserved across inteins found in different organisms. The splice junctions are indicated by arrows, while conserved amino acids involved in the splicing mechanism are boxed (see also Hirata et al., 1990; Kane et al., 1990; Davis et al., 1992; Hodges et al., 1992).

In the early 1990s, Protein A affinity chromatography was a well-established platform for the purification of monoclonal antibodies (mAbs) (Bolton and Mehta, 2016). The success of Protein A chromatography in purifying different mAbs prompted the search for a similar platform technology that could purify non-mAb proteins irrespective or size, structure, and sequence. It was soon recognized that inteins could be used in conjunction with conventional affinity tag methods to potentially provide this platform, where the intein could provide a means for self-removal of the affinity tag under controlled conditions (Chong et al., 1997). Most of the early work in developing inteins as a protein purification platform was done by researchers at New England Biolabs (NEB), where thermophilic inteins were used to elucidate the canonical splicing mechanism and related active-site amino acids. In one of the first intein patents (Comb et al., 1996; Patent No.: US 5496714 A; filed in 1992, approved in 1996 and expired in 2013), scientists at NEB described methods to synthesize modified proteins comprising a target protein and Controllable Intervening Protein Sequences (CIVPSs) that could splice in either a *cis* (contiguous intein) or *trans* (split intein) configuration. The CIVPSs were “controllable” because their splicing or cleaving reactions could be induced by predetermined conditions such as exposure to light or a change in temperature. The patent also discussed methods for inserting an intein into a target gene by making silent mutations near the ends of the intein or by creating suitable restriction sites in the target gene. Perhaps most importantly, this patent also broadly defined inteins as CIVPSs and identified them based on specific conserved features (Figure 2), thereby providing prior art that would apply to new intein sequences that had not yet been discovered and many of their applications (Hodges et al., 1992).

The methods described by this patent had revolutionary implications in protein engineering. One immediate suggestion was that if the CIVPSs were properly placed, they could reversibly and controllably inactivate a toxic protein. In another embodiment they could be used to construct large protein complexes by posttranslational *trans*-splicing of protein segments using split inteins. Notably, this patent also suggested that CIVPSs could be manipulated for recombinant protein purification via the generation of self-cleaving affinity tags.

**FIGURE 3:**
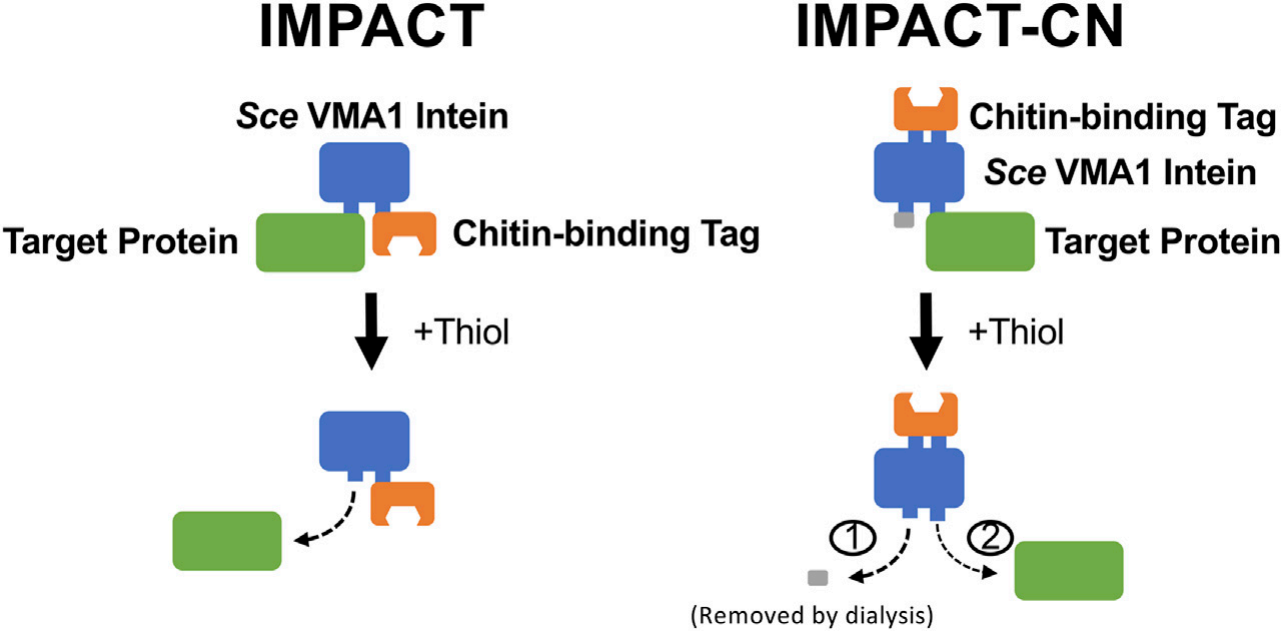
Schematics of the IMPACT and IMPACT-CN systems developed by New England Biolabs (Chong et al., 1998; Wood & Camarero, 2014). One-step purification of a tagless target protein by a self-cleaving chitin-binding domain tag is enabled by a modified *Saccharomyces cerevisiae* VMA1 intein in the IMPACT system (Comb et al., 1998; Patent No.: US 5834247 A; given priority in 1992, issued in 1998 and expired in 2012). N-terminal intein cleavage was induced by addition of 30 mM DTT (thiol) at pH 7.6 and 4°C and required an incubation period of 16 h. The IMPACT-CN system allows for either N- or C-terminal cleavage, with an extra dialysis step required for C-terminal cleavage (Comb et al., 1998; Patent No: US 5834247 A; given priority in 1992, issued in 1998 and expired in 2012), showed that separation and purification of target proteins can be improved by inserting the affinity tag into a modified *Sce* VMA1 intein. In practice, both N- and C-terminal cleavage reactions are induced while the fusion protein is immobilized on the column, and the small N-terminal segment is later removed by dialysis.

Given these extraordinary possibilities, an additional broad patent was soon after filed by NEB in 1997 (Comb et al., 1998; Patent No.: US 5834247 A; given priority in 1992, issued in 1998 and expired in 2012). In this patent, NEB scientists described methods for purifying proteins by using a “three-part fusion” comprising an intein, target protein and a conventional affinity tag. Cleavage was extremely specific to the intein-POI junction and the patent claimed that cleavage could be induced by changing the system temperature, varying the solution conductivity, adding chemical reagents, or changing the solution pH; each of which could potentially result in a highly purified and tagless target protein. One embodiment of the claims included the 454 amino acid *S. cerevisiae* VMA1 intein (New England Biolabs, n.d.) and a chitin binding domain (CBD) as the affinity tag, where the target protein is released via thiol-dependent N-terminal intein cleavage. This embodiment was later commercialized as the Intein Mediated Purification with an Affinity Chitin-binding Tag or IMPACT system (Figure 3). The patent also described the use of inteins with an immunoaffinity approach, where an affinity resin with antibodies specific to the intein would be used in place of an affinity tag.

The IMPACT system was especially effective due to the strong affinity between the CBD and corresponding chitin affinity resin, which was sold as a consumable reagent by NEB. Cleavage in the original IMPACT system was induced by addition of up to 50 mM thiol, which cleaves the thioester bond that forms during step 1 of the splicing reaction. In this case, the intein has been modified such that it cannot progress through the splicing reaction, thus stabilizing the thioester for chemical cleavage by thiol or other strong reducing compounds. Examples in the patent describe the expression of intein fusion proteins in both yeast (*S. cerevisiae*) and bacterial (*E. coli*) expression systems, and effectively demonstrated the versatility of the system. Further, in contrast to traditional affinity chromatography, restriction sites were available in the IMPACT system plasmids that allowed target protein cloning and purification with no additional vector-derived amino acids on the purified POI. The purification of various model proteins including the restriction endonuclease FseI, green fluorescent protein (GFP), calmodulin-dependent protein kinase (CamKII), *S. cerevisiae* invertase, rabbit protein phosphatase-1 (PP1) and T4 DNA ligase have been demonstrated using the IMPACT system (Chong et al., 1998).

Although the original IMPACT system only provided N-terminal intein cleavage (where the target protein is cloned upstream of the intein), the subsequent IMPACT-CN system allowed for either N or C-terminal cleavage, with an extra dialysis step required for C-terminal cleavage (Figure 3). The currently available IMPACT-CN system includes convenient pTYB expression vectors (used for cloning and recombinant protein expression in *E. coli*), which allow fusion of the intein tag to either the N-terminus or C-terminus of the POI. The versatility of the IMPACT-CN system significantly increases the probability of successful POI expression and purification compared to the IMPACT system.

In the IMPACT-TWIN system, the target POI sequence is cloned between two mini-inteins, where the vector is designed to produce a POI with a specific intein fused to either its N or C-terminus. Each intein is in turn fused to a chitin binding domain, thus allowing protein purification on chitin resin via C or N-terminal intein cleaving. The *Synechocystis* sp. DnaB mini-intein fused to the N-terminus of the POI has been engineered to undergo pH and temperature-dependent C-terminal cleavage. The other mini-intein used is either the *Methanobacterium thermoautotrophicum* RIR1 (pTWIN2) or the *Mycobacterium xenopi* GyrA (pTWIN1) mini-intein. Both of these mini-inteins have been engineered to undergo thiol-induced N-terminal cleavage. Elution of the POI from the chitin resin is induced by cleavage of the fused mini-intein, while the intein remains bound to the resin through the chitin binding domain (New England Biolabs Inc., 2008).

These NEB patents demonstrated that inteins could achieve single-step purification of native target proteins without the need for a protease to remove the affinity tag. This success sparked broader interest in inteins among both academicians and scientists at biotech companies, who began developing additional self-cleaving tag purification systems. However, significant roadblocks remained. The IMPACT and pTWIN systems exhibited variable performance with regard to cleaving kinetics, and cleavage of the purified POI could be unacceptably slow. Moreover, the N-terminal cleaving inteins used in the NEB systems require thiol-containing chemical reagents like DTT, β-mercaptoethanol or free cysteine for cleavage, which was found to be undesirable for scaled-up operations due to high cost and potential toxicity. The required addition of thiol reagents also presents problems for target proteins having disulfide bonds due to their tendency to break these bonds and disrupt the integrity of the target protein. The C-terminal cleaving inteins, which are controlled by pH and temperature, eliminated the need for thiols, but instead exhibited target-dependent premature cleavage before the purification step. Furthermore, the *S. cerevisiae* VMA1 intein used in the IMPACT system, with a size close to 56 kDa, is often significantly larger than the target proteins being purified, which decreases expression efficiency of the POI. Finally, when the IMPACT-CN system is used for C-terminal intein cleaving, a small peptide of about 1.2 kDa cleaves from the intein tag and elutes alongside the POI, requiring removal by dialysis (New England Biolabs, n.d.).

## The Second-Generation Patents Tried to Come up With Better Intein and Cloning Systems

The patents that followed the original NEB patents describe better contiguous inteins with more controllable and efficient cleaving properties. Rational design and directed evolution approaches played a crucial role in this endeavour. A subsequent NEB patent (Perler and Adam, 2007; Patent No.: US 7157224 B2; prioritized in 1992, filed in 2002, approved in 2007 and expired in 2014) claimed the development of positive genetic selection systems to screen for mutations or external agents that can either inhibit or activate intein splicing or cleaving. These methods were used to identify temperature sensitive inteins via evolutionary methods based on error-prone polymerase chain reaction (PCR) or insertion of combinatorial DNA sequences at specific regions within the intein. Highly specific mutations were also made to residues thought to influence or assist splicing or cleaving, such as residues close to the intein splice junctions or within the conserved intein Block B (Perler et al., 1997), or residues determined to be proximal to the intein active site (Duan et al., 1997). The mutated intein genes were then introduced into cells and examined for the ability to controllably splice under desired conditions. The patent also discusses methods for generating temperature-controlled inteins and demonstrated their utility by developing temperature sensitive *Mxe* GyrA intein mutants. In one version of this intein, N-terminal cleavage was induced using thiol reagents, while C-terminal cleavage was induced by a temperature shift from 37°C to room temperature (16–25°C) (Southworth et al., 1999).

**FIGURE 4:**
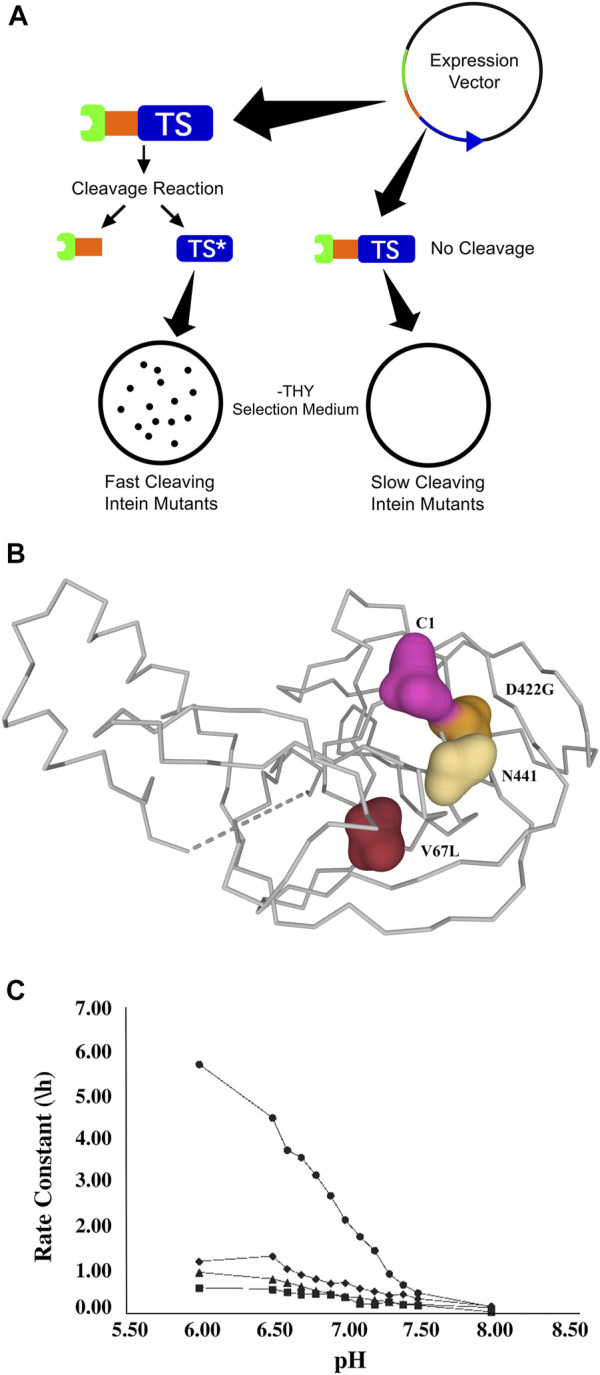
**(A)**. The thymidylate synthase (TS) reporter system that was central to a patent filed by scientists at RPI and New York State Health Department (Belfort et al., 2005; PCT Application No.: WO 2001/012820 A1, Patent No.: US 6933362 B1; prioritized in 1999, filed in 2000 and approved in 2005). A plasmid was constructed to overexpress a tripartite fusion of maltose binding protein/intein/TS. The basis of the selection system is that the tripartite fusion has no TS activity, while C-terminal intein cleavage yields active TS and a resulting selectable growth phenotype. This tripartite reporter is therefore useful for the selection of controllably cleaving inteins. **(B)**. The patented mutations that affect the splicing or cleaving activity of the engineered ∆I mini-intein, superimposed onto the *Mxe* GyrA intein structure (PDB ID 1AM2, Klabunde et al., 1998, Berman et al., 2000). It was hypothesized that in the wild-type intein, electrostatic interactions between a conserved Asp (D422) and C-terminal Asn residue inhibit the C-terminal cleaving step of the splicing reaction until after extein ligation. The D422G mutation removes this interaction and thus facilitates C-terminal cleavage in the absence of extein ligation. The additional V67L mutation confers stability on the mini-intein created after the deletion of the homing endonuclease region, allowing native-like splicing activity to be restored in the mini-intein. C1 and N441 indicate superimposed locations of N-terminal and C-terminal intein residues, respectively. **(C)**. This figure shows the effect of pH on cleavage activity of the mini-intein mutants discussed in the patent. The plotted rate constants are for cleavage of tagged target protein to products at 37°C and the indicated pH (Wood et al., 1999). In the figure, ◆ is the full-length wild-type intein, ▪ is the rationally engineered ΔI mini-intein, * is the ΔI mini-intein with the V67L mutation, and • is the ΔI mini-intein with the D422G mutation (referred to as ∆I-CM). It was observed that cleavage rates increased as the pH was reduced, typically increasing by a factor of 8 or more as the pH was decreased from 8.0 to 6.0. The strongest pH activation was exhibited by the ΔI-CM intein, for which the cleavage rate increased by a factor of more than 20 over this pH range. The cleavage inhibition at high pH was reversible in all cases, allowing tripartite precursor to be stored for several days at a temperature of 4°C and at pH 8.5 without significant cleavage or loss of activity (Wood et al., 1999).

A similar patent (Belfort et al., 2005; PCT Application No.: WO 2001/012820 A1, Patent No.: US 6933362 B1; prioritized in 1999, filed in 2000 and approved in 2005), filed by scientists at the Rensselaer Polytechnic Institute (RPI) and the New York State Department of Health, describes efforts to address three major issues affecting the NEB inteins: cleavage rate, cleavage controllability and intein size (Figure 4). This patent focuses primarily on engineered modifications to the *Mycobacterium tuberculosis* recA intein. To address the large size of the intein, the intein’s homing endonuclease domain was deleted, resulting in a decrease in size from 440 amino acids to 168 amino acids with some loss of activity. The resulting engineered mini-intein was then subjected to mutagenic PCR and genetic selection for greater splicing or cleaving activity via a thymidylate synthase selection system (Figure 4A). One of the resulting inteins, now referred to as ΔI-CM, was shown to have pH and temperature dependent cleavage, with highly accelerated C-terminal cleavage at lower pH and elevated temperatures (Wood et al., 1999). The C-terminal cleavage activity exhibited by this intein is not coupled to N-terminal cleavage (as with the earlier IMPACT-CN system), and thus is not dependent on thiol compounds or other reducing agents. Further, the ∆I-CM mini-intein exhibits 20-fold–40-fold higher activity at pH 6.0 than at pH 8.5 (Figure 4C), allowing good control of cleaving within a fairly mild pH range over the course of a purification. The patent also describes the specific mutation (a highly conserved aspartic acid to glycine, Figure 4B) that provides the accelerated cleaving phenotype and suggests that homologous mutations may be used in other inteins to achieve similar results based on a proposed mechanism for splicing regulation. The use of this intein in a modified affinity separation method has now been demonstrated for several proteins.

In another patent application (Wood, et al., 2009; Patent Application No.: US 2009/0098611 A1; prioritized in 2004, filed in 2005 and subsequently abandoned in 2009), scientists at Princeton University addressed the need to develop more efficient *in vitro* and *in vivo* cloning systems for use with the ∆I-CM mini-intein. The invention listed nucleotide sequences encoding modified inteins having enhanced cleavage activity, where these sequences could be combined with topoisomerase recognition sequences at suitable sites to enable topo-cloning. The Topo, Gateway and Topo + Gateway inteins were generated by modification of the original *Mtu* RecA ΔI-CM mini-intein, where the resulting system would allow Topo and Gateway cloning with various combinations of promoters, affinity tags and inteins in order to accelerate the cloning and characterization of individual proteins (Gillies, et al., 2008; Wu, et al., 2010).

## Third-Generation Patents Focus on the Use of Split Inteins Over Contiguous Inteins

Most reported split inteins, whether naturally occurring or artificially engineered, are divided between the conserved intein motifs B and F (Mills et al., 2014). In contiguous inteins, this location typically contains the intein homing endonuclease domain, which is thought to provide a means for inteins to spread within and between genomes. Naturally occurring split inteins normally lack a homing endonuclease domain, and like their engineered counterparts they typically consist of a larger N-terminal fragment of over 100 amino acids and a smaller C-terminal fragment of 25–40 amino acids (Saleh and Perler, 2006). A recurring problem with artificially split inteins has been misfolding of the tagged targets due to instability of the intein segments. This observation led to efforts to decrease the size of one or the other intein segment through rational engineering and truncation. These efforts met with mixed success, where it was discovered that heavily truncated inteins often lose binding or splicing/cleaving activity. A notable example of this approach was an attempt to generate a six amino acid long C-terminal intein segment from the *Ssp* DnaB split intein (Sun et al., 2004).

**FIGURE 5:**
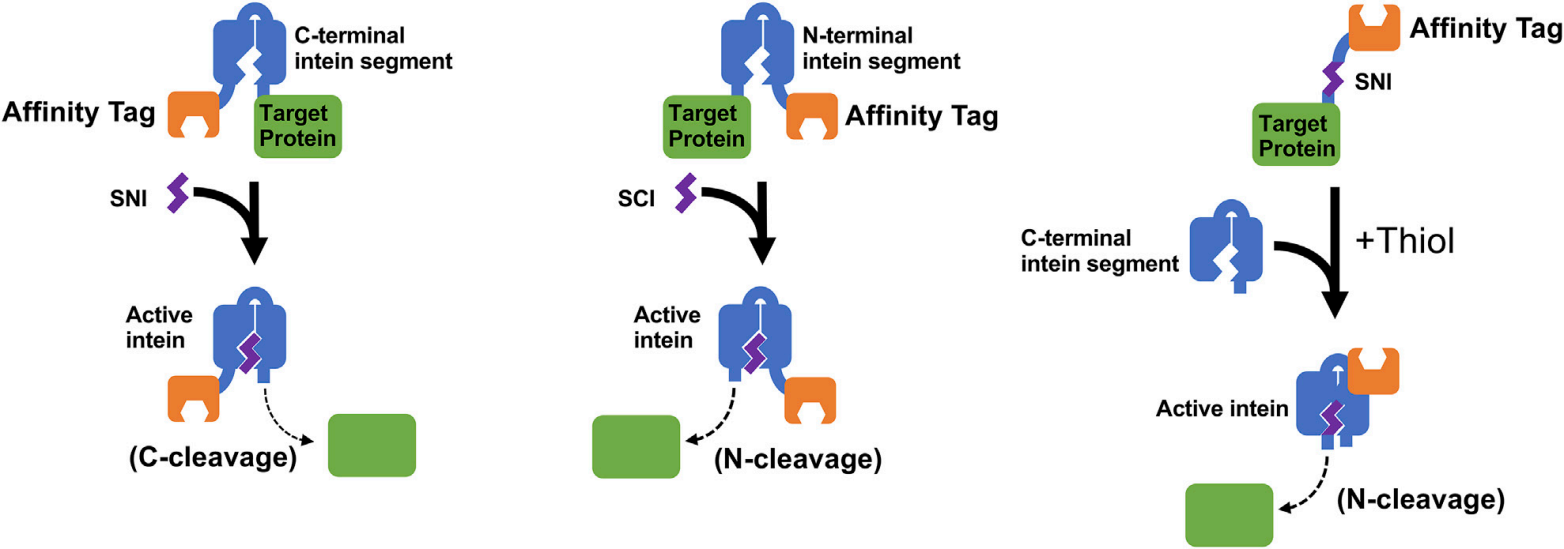
This figure illustrates schemes of controllable protein cleavage using the SCI and SNI split inteins discussed in a patent filed by scientists at Dalhousie University (Liu et al., 2013; Patent No.: US 8394604 B2; prioritized in 2008, filed in 2009 and approved in 2013). The recombinant precursor protein typically consists of a Target Protein, either the N-terminal intein segment or C-terminal intein segment, and an optional Affinity Tag for easy purification of the precursor protein. Cleavage at the intein’s C-terminus or N-terminus can be triggered by the addition of the complementing Small N-intein (SNI) or Small C-intein (SCI), respectively, or by addition of the C-terminal intein segment to a smaller SNI Affinity Tag fusion as shown in the figure (Volkmann et al., 2009).

Despite these difficulties, scientists at Dalhousie University filed a patent (Liu et al., 2013; Patent No.: US 8394604 B2; prioritized in 2008, filed in 2009 and approved in 2013) claiming the discovery of methods to make small functional N/C intein fragments. Although this patent focused primarily on splicing applications with split inteins, the inventors also demonstrated controllable cleavage using **S**mall **N/C I**ntein fragments and used these fragments (SNI and SCI) to perform protein purifications (Figure 5). Interestingly, the N-terminal cleavage system developed using this method showed greater cleavage efficiency (∼95%) than the IMPACT system (albeit in the presence of 100 mM DTT), while the C-cleavage system had comparable cleavage efficiencies (∼88%) to systems developed from other contiguous inteins. It was speculated that the small size of the intein fragments resulted in reduced protein misfolding and better protein expression.

A significant discovery was subsequently made by (Zettler et al., 2009), which described robust and extremely fast protein splicing kinetics of the naturally split *Npu* DnaE intein. A notable characteristic of this intein is its ability to splice efficiently in the presence of very diverse flanking extein sequences, while the split intein segments were also much more soluble during expression due to this being their native state. This was a major step forward since most inteins up to that point had exhibited a strong preference for their native flanking extein residues, which required the cleaved target proteins to have added extein amino acids (or “scars”) when using those inteins for purification. The ability to efficiently cleave unmodified targets, along with demonstrated faster kinetics was a key driver for a shift towards the development of split intein based purification technologies. Another driver was the fact that premature cleavage of target proteins during expression was not an issue with split inteins since cleavage only takes place once the two fragments are contacted and assemble under suitable conditions.

The first successful split intein patent (Bergwerf et al., 2018; PCT Application No.: WO 2013/045632 A1, Patent No.: US 10100080 B2; prioritized in 2011, filed in 2012 and approved in 2018) was filed by scientists at ERA Biotech (Barcelona, Spain) which was later acquired by ZIP Solutions, SL. The patent claimed the development of robust split inteins that could operate over a large range of pH and temperature and could even function reasonably well in the presence of chaotropic salts. The specific split inteins named in the patent, NrdJ1, gp41-1, gp41-8 and IMPDH1, were also found to have higher splicing rates than the natural *Npu* DnaE split intein. While the *Mtu* RecA and *Ssp* DnaE split inteins showed reduced yields and a higher proportion of unwanted cleavage side products at 37°C, gp41-1 was found to have its highest efficiency at that temperature. It also spliced more efficiently than *Npu* DnaE, with 90–95% of the spliced product forming within 5 min in one example. Once converted to a C-terminal self-cleaving intein by mutation of the initial Cys residue to Ala, these inteins were also tested for purification applications. While most RecA inteins, including ∆I-CM have an optimal cleaving range between pH 6 and 7.5, gp41-1 was found to be independent of pH between pH 6 and 9, and only showed a significant drop in activity at extreme pH values of 4 and 10. Furthermore, while the N-terminal self-cleaving reaction was very inefficient for these split inteins, the C-terminal cleaving reaction occurred even when the five native flanking C-extein amino acids were absent. These characteristics made these inteins highly attractive for protein purification applications.

**FIGURE 6:**
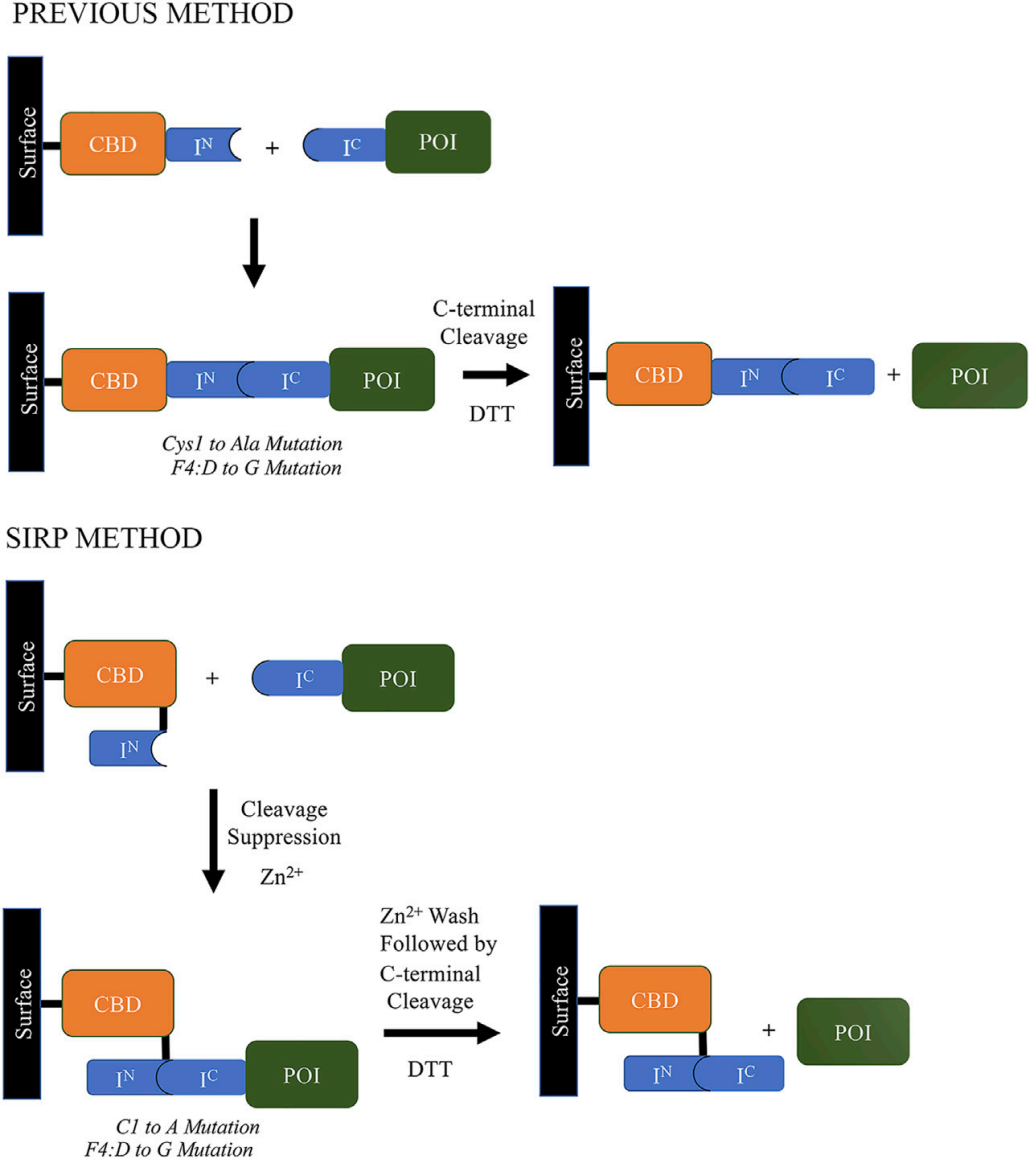
Comparison of the Split Intein mediated ultra-Rapid Purification system (SIRP) developed by scientists at Texas A&M University (Chen et al., 2018; Patent No.: US 10087213 B2; prioritized in 2013, filed in 2014 and granted in 2018) vs already existing intein-based cleaving method at that time, which was used in labs (Guan et al., 2013; Guan & Chen, 2017; Wood et al., 1999).

Soon thereafter, scientists at Texas A&M University claimed the development of an engineered *Npu* DnaE split intein that provided extremely fast cleaving under specific conditions. The system, referred to as the Split Intein mediated ultra-Rapid Purification system or SIRP, is described in their patent (Chen et al., 2018; Patent No.: US 10087213 B2; prioritized in 2013, filed in 2014 and granted in 2018), and claims the same conserved aspartic acid to glycine mutation that was reported in the ∆I-CM mini-intein, which similarly accelerated isolated C-terminal cleaving (Wood et al., 1999; Guan and Chen, 2017). Further, a new configuration was also disclosed (Figure 6), wherein the purification tag was relocated to the C-terminus of the N-terminal intein segment, resulting in highly improved C-terminal cleavage. The result was reported to be ∼80% cleavage yield in 3 h at 22°C, which is much better than the IMPACT system’s 16 h at 23°C for a similar yield. However, the split intein system described in this patent still requires significant concentrations of thiol reagents to induce efficient cleavage.

At the same time, scientists at Princeton University were looking for better ways of making and isolating protein α-thioesters (Muir et al., 2020b; Patent No.: US 10,526,401 B2; prioritized in 2012, filed in 2017 and granted in 2020). In this approach, a modified *Npu* DnaE intein is used to purify recombinant protein α-thioesters and label them, so they can be used as tools to study biological processes in cells.

**FIGURE 7:**
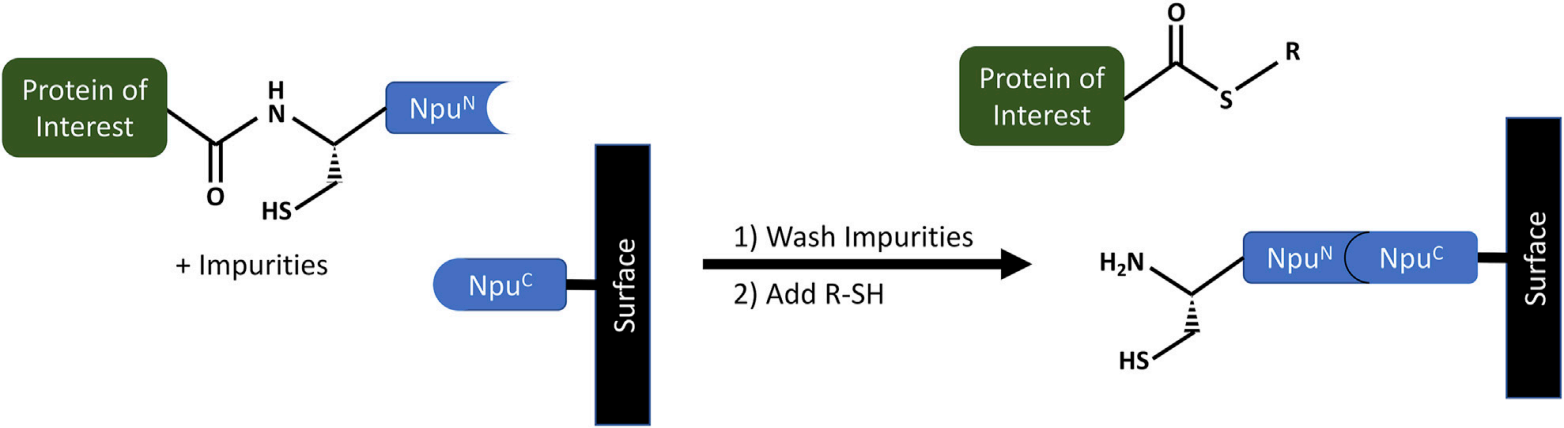
Purification of recombinant protein alpha-thioester using the Npu^C^-AA affinity resin developed by a team at Princeton University (Muir et al., 2020b; Patent Application No.: US 10,526,401 B2; prioritized in 2012, filed in 2017 and granted in 2020). The Npu^N^ portion of a modified split *Npu* DnaE intein is genetically fused to the Protein of Interest (POI). During the loading step it spontaneously binds to the Npu^C^ intein fragment immobilized on the affinity resin. After a wash step to purify the POI, a thiolysis reaction is triggered to cleave the POI by use of a thiol buffer. The POI is cleaved and eluted to be later used for fluorescent labeling via a Cys-containing short peptide.

The process incorporates a purification method, which follows standard purification steps of load, incubate, wash and elute, but where the eluted protein is conjugated to a specific ligand via an α-thioester (Figure 7). The purification relies on an Npu^C^-AA mutant, where Asn^137^ and Cys^+1^ amino acids of a wild-type Npu^C^ are both replaced by Alanine and immobilized on agarose SulfoLink^TM^ resin (Pierce) as an Npu^C^-AA-Cys-OMe peptide. At the same time, the POI is genetically fused to the N-terminus of the Npu^N^ fragment of the intein. In practice, the Npu^N^ and Npu^C^ fragments spontaneously bind to each other on the column, generating a reconstituted intein while capturing the tagged POI, which is then purified by a washing step. After the impurities are removed, a buffer containing thiol (R-SH), such as mercaptoethansulfonate (MES), is used to trigger a thiolysis reaction leading to POI cleavage. The eluted POI is a protein α-thioester, where the R-group of the thiol is conjugated to the C-terminus of the POI. The patent provides several examples, including a ubiquitin-α-thioester and a maltose binding protein-α-thioester. The RP-HPLC and MS analysis showed that obtained recovery yields were between 75–95%, where the loading capacity of Npu^C^-AA resin was 3–6 mg of protein/mL. The total isolated yields were reported to be 2.5 mg/L of *E.coli* culture for Ub-MES and 40 mg for MBP-MES (Vila-Perelló et al., 2013).

Additionally, other semi-synthetic protein complexes were made for more challenging proteins. In one important example, the authors purified a fragment of histone H2B (residues 1-116) by expressing H2B-Npu^N^ in *E.coli* cells. During purification, the H2B-Npu^N^ was loaded on the column in 2 M urea and incubated at pH 6.0 for 3 h, followed by a change to pH 7.2 to optimize the thiolysis reaction in the presence of MES (36 h at room temperature). The H2B-MES was obtained with a high purity of >90% and isolated yield of ∼20 mg/L of culture, compared to 4 mg/L of culture obtained using other methods at that time. The purified H2B-MES was then modified using H-CVTK(Ac)YTSAK-OH ligation to produce histone H2B acetyl Lys120 (H2B-K120Ac). They also optimized α-DEC205 α-thioester production by using a wider range of ultra-fast split inteins (*e.g.* Ava, Csp, Cwa, Mcht, Oli, Ter, and Cra) to form and test different prototypes such as α-DEC205-Ava^N^ and α-DEC205-Npu^N^. However, the Ava^N^ and Csp^N^ intein fusions showed higher expression levels compared to other tested split inteins. As a result, they were able to obtain αDEC205-MES, which later was modified to contain a fluorescent peptide CGK(Fl). By using an α-DEC205-Ava^N^ fusion, the αDEC-CGK(Fl) was obtained at 75% yield.

## Recent Patents Have Tackled Purification System Performance Issues

**FIGURE 8:**
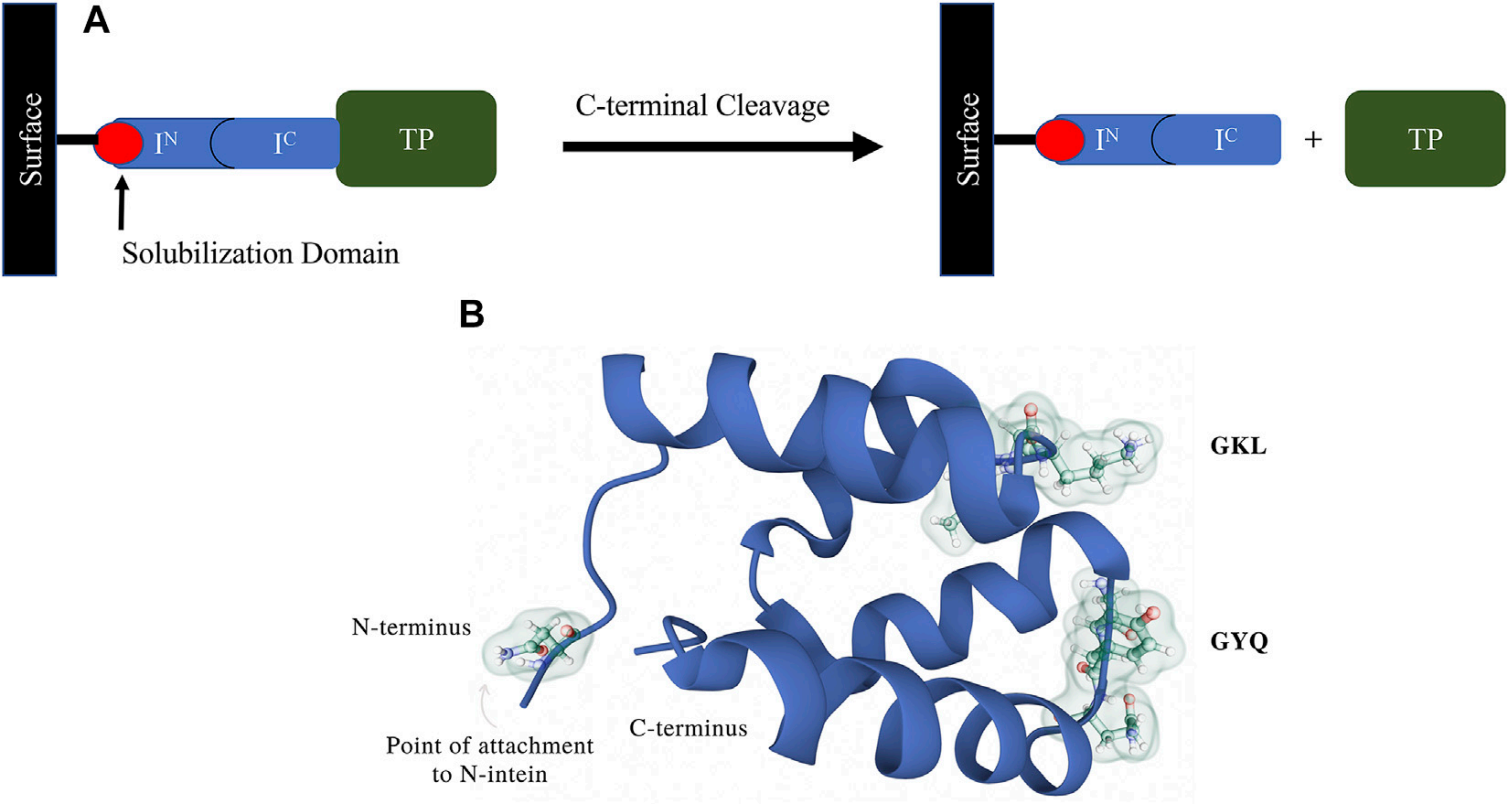
**(A)**. This figure from a patent filed by scientists at Merck KGaA (Zillmann and Orlando, 2019; Patent No.: US 10308679 B2; prioritized in 2014, filed in 2015 and approved in 2019) presents a schematic diagram depicting an affinity purification method based on a self-cleaving tag, as well as a solubilization domain used for manufacturing the split intein ligand. The method employs an affinity chromatography matrix listed in the invention comprising a fusion protein having an N-intein polypeptide fused to an N-intein solubilization partner that is attached to a solid support. A second fusion protein comprising a C-intein that is complementary to the N-intein on the affinity matrix is fused to the target protein to be purified (POI) alongside any other elements required for expression, such as secretion signals. **(B)**. The structure of solubilization partner 138 (p138, PDB ID 1RYK) is also shown (Yee et al., 2002). The protein contains four alpha helix domains, is globular and has a long unstructured coil that forms the connection to the carboxy terminus of the N-intein. The loop regions GKL and GYQ were targeted for cysteine residue insertions away from the catalytic site to create the new versions of p138 that would be functionalized for single-point resin attachment via thiol-coupling chemistry.

Patents published within the last 6 years have tried to find solutions to problems associated with intein resin development and manufacturability. While split inteins offer significant advantages over contiguous inteins as purification platforms, the ability to manufacture a practical intein resin has been hampered by poor solubility and aggregation of N-terminal intein fragments. These N-terminal intein segments act as the affinity ligand for the intein system and must be produced and immobilized efficiently in order to produce a cost-effective intein affinity resin for commercial use. To address these issues, scientists at Merck KGaA (Darmstadt, Germany) designed a fusion protein comprising a solubilization partner, with a molecular weight less than 15 kDa, joined to an N-terminal intein segment. The solubility partner also provides a means of coupling the fusion protein to a resin backbone (Figure 8A). These scientists claimed in their patent (Zillmann and Orlando, 2019; Patent No.: US 10308679 B2; prioritized in 2014, filed in 2015 and approved in 2019) that the inclusion of the solubilization partner (Figure 8B) increased the solubility of the fusion protein by up to 90% from the original N-intein alone. This invention thereby solved a major issue associated with N-intein insolubility and rendered their inteins suitable for the production of a cost-effective intein affinity resin.

**FIGURE 9:**
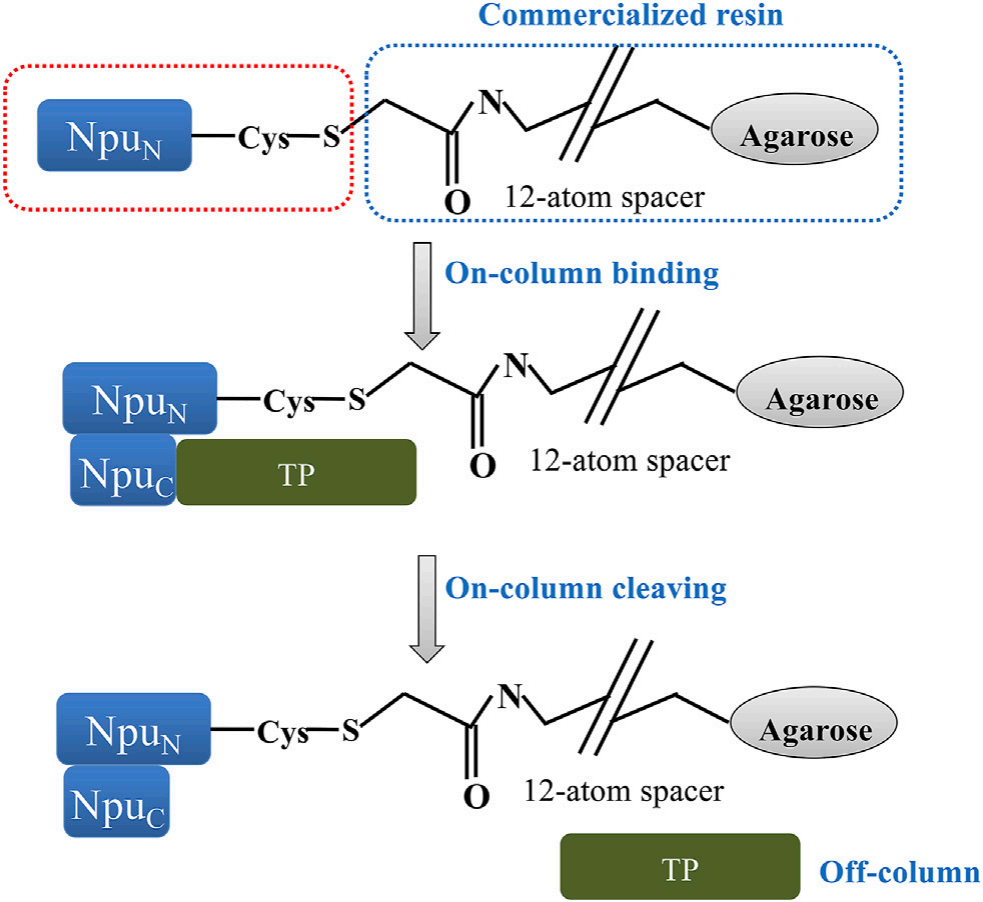
This figure was adapted from a patent filed by scientists at the Ohio State University (Wood and Shi, 2018; Patent No.: US 10066027 B2; prioritized in 2015, filed in 2016 and approved in 2018, with continuation Patent No.: 10669351 B2) and shows a scheme of split intein immobilization and the corresponding purification method. The N-terminal intein segment (Npu_N_) is expressed and purified from a recombinant protein expression host (in this case *E. coli*), and then immobilized onto a commercially available immobilization resin (e.g., SulfoLink™ resin). The charged resin can be used to directly capture the intein C-terminal intein segment (Npu_C_), which has been fused to a Target Protein (TP). After the feed impurities have been washed away, the intein can be induced to release the target protein (TP) from the column matrix by a pH or temperature shift. The cleaved tag-free target can be directly collected from the column, and the column can be regenerated to remove the cleaved intein C-terminal segment, allowing multiple rounds of purification on a single batch of resin.

**FIGURE 10:**
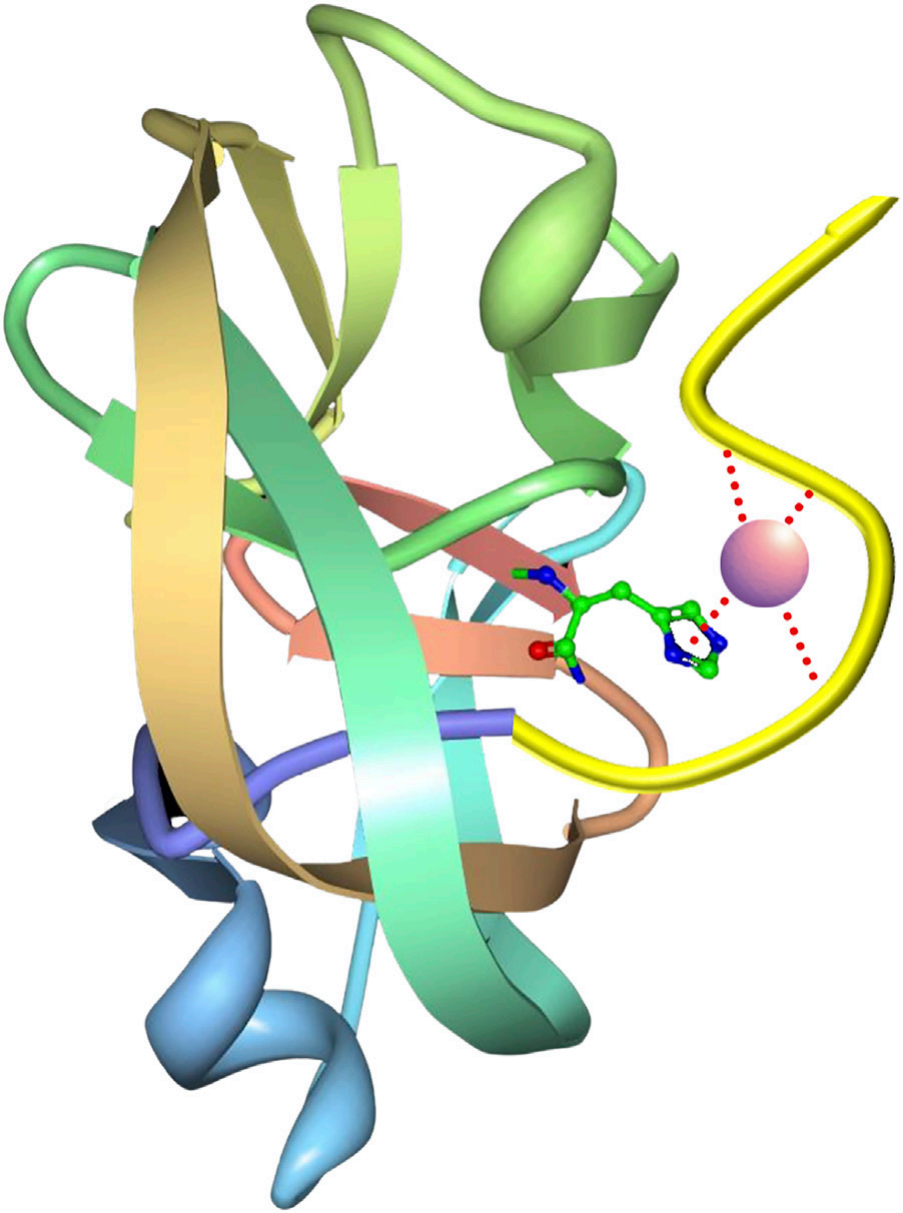
Ribbon diagram of the ΔI-CM mini-intein with an engineered N-terminal zinc binding motif and bound zinc ion interacting with a conserved His residue (ball-and-stick) close to the C-terminal cleaving junction. If properly designed, this motif will greatly increase the affinity for zinc by the intein, allowing a much higher sensitivity to zinc for suppressing cleaving during expression and purification of a fused target protein. The resulting intein is described in the associated patent (Ma et al., 2019; Patent No.: US 10323235 B2; prioritized in 2012, filed in 2017 and approved in 2019). The structure of PDB ID 2IN0 is here presented (Van Roey et al., 2007).

An additional split intein patent filed by scientists at the Ohio State University (Wood and Shi, 2018; Patent No.: US 10066027 B2; prioritized in 2015, filed in 2016 and approved in 2018, with continuation Patent No.: 10669351 B2) claims the development of an engineered *Npu* DnaE purification system with a covalently immobilized N-terminal intein fragment (ligand) and a self-cleaving C-terminal intein fragment (affinity tag) attached to a target protein (TP, Figure 9). Test TPs were purified using the system, including streptokinase, secreted embryonic alkaline phosphatase (SEAP; a glycoprotein containing disulfide bonds) and human granulocyte colony stimulating factor (G-CSF). Interestingly, it was observed that fusion of SEAP with the intein tag did not abolish glycosylation. The purification scheme depends on a pH 8.5 buffer for inhibiting the C-terminal cleaving reaction during purification, and a pH 6.2 buffer for inducing the cleaving reaction following purification. To achieve greater sensitivity to pH and zinc ion concentration, a novel zinc binding motif was introduced to the immobilized N-terminal intein fragment. This motif was described further in an additional patent (Ma et al., 2019; Patent No.: US 10323235 B2; prioritized in 2012, filed in 2017 and approved in 2019) by scientists at the Ohio State University and the National Cancer Institute. The rationally designed zinc binding motif was appended to the N-terminus of the intein and was intended to pull the active site histidine into a metal chelation arrangement, making C-terminal peptide bond cleavage difficult in the presence of zinc ion (Figure 10). Surprisingly, the zinc binding motif introduced enhanced pH sensitivity to the intein as well. This intein and the corresponding affinity resin has now been commercialized as the *i*CapTag™ system by Protein Capture Science, LLC.

At about the same time, scientists at GE Healthcare Bioprocess R&D (now Cytiva Bioprocess R&D AB, Uppsala, Sweden) filed a patent application (Ohman et al., 2019; U.S. Patent No. 11,124,539 B2; prioritized in 2016, filed in 2017 and granted in 2021) that claims the development of an improved chromatography resin based on the split *Npu* DnaE intein. This patent describes covalent immobilization of the N-terminal intein fragment and provides high ligand densities with the potential to reuse the resin after multiple harsh cleaning procedures. A general protocol for manufacturing the resin describes expression of the N-terminal split intein fragment in insoluble inclusion bodies within bacterial cells such as *E. coli*. The cells are subsequently harvested and disrupted, at which point the inclusion bodies are solubilized and contacted to an IMAC or ion-exchange resin to promote column-assisted refolding. The refolded N-terminal intein fragments are then contacted with pre-activated porous polymer beads under suitable conditions for covalent attachment. The patent demonstrates a successful split intein purification of green fluorescent protein (GFP) using a prepared column.

**FIGURE 11:**
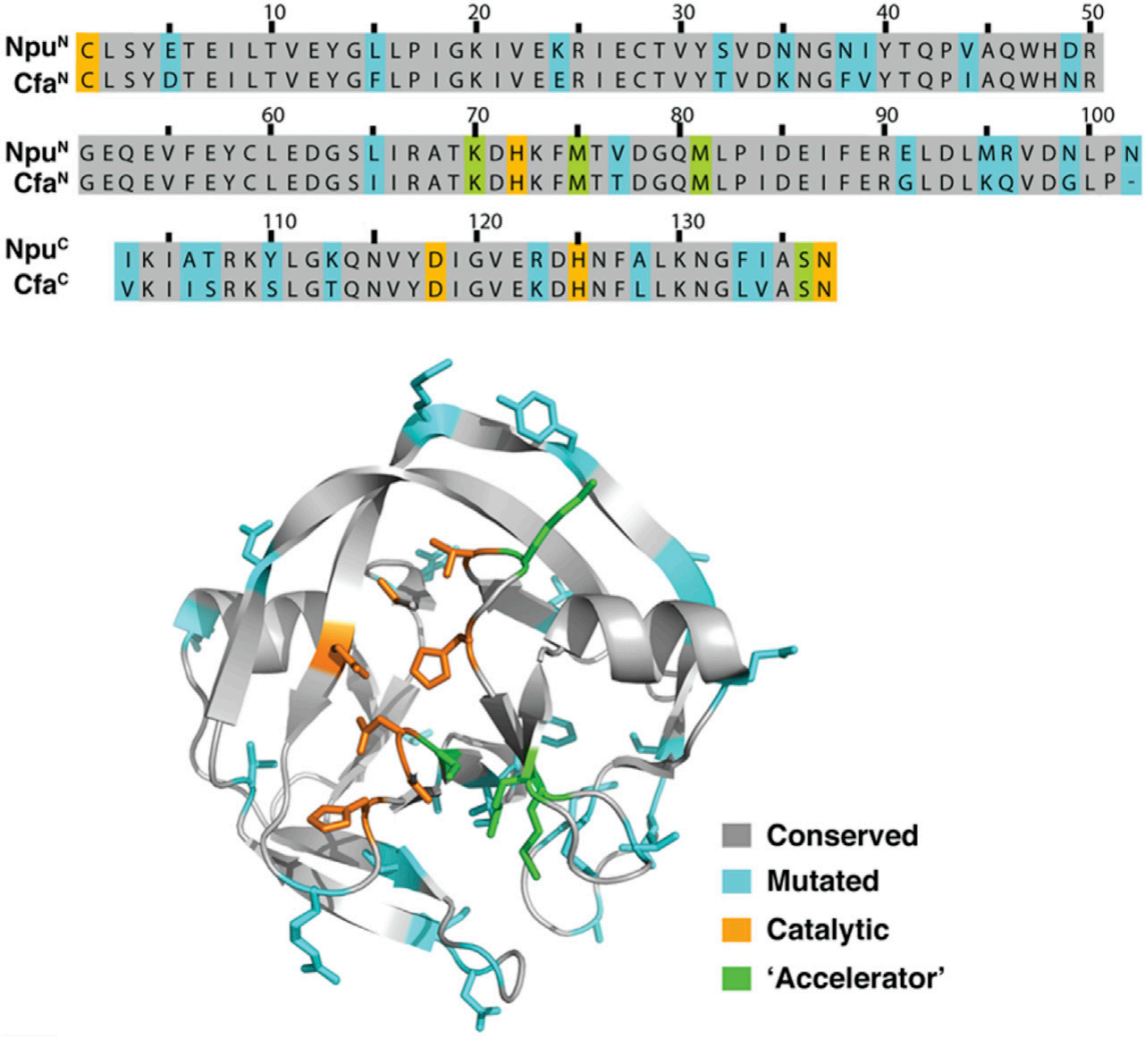
Comparison of the sequence alignment of *Npu* DnaE intein (PDB ID 4KL5) and engineered Cfa split intein presented by scientists at Princeton University (Muir et al., 2020a; Patent Application Publication No.: US 2020/0055900 A1; prioritized in 2016 and filed in 2017). A computer-generated model of the design of the Cfa split intein, according to an embodiment of the invention, has also been shown, where the general effects of various mutations are indicated (Aranko et al., 2013; Stevens et al., 2016; Gramespacher et al., 2018). Reproduced with ACS permission from Stevens et al., 2016.

In a separate approach to solve the problem of N-intein fragment aggregation, scientists at Princeton University used “consensus protein design” on the family of DnaE split inteins. This approach led to the development of Cfa^N^ and Cfa^C^ intein fragments that can perform protein trans splicing (PTS) at high temperatures and in the presence of chaotropes (Gramespacher et al., 2018). Interestingly, they observed that exteins fused to Cfa fragments had better expression yields than exteins fused to natural *Npu* DnaE fragments in both mammalian and bacterial cells. In a patent (Muir et al., 2020a; Patent Application No.: US 2020/0055900 A1; prioritized in 2016 and filed in 2017), it was further revealed that the Cfa intein had 82% sequence similarity to the *Npu* DnaE split intein (Figure 11) and was found to splice 2.5 times faster than the *Npu* DnaE split intein.

## Conclusion and Future Perspective

Inteins have undergone a lot of changes from the days when they were first identified. Once a simple curiosity for the scientific community, inteins were soon recognized as potential protein engineering and purification platforms. Despite some limitations of early systems, research continued and now several patents have been filed that address most of the roadblocks to the commercialization and scale up of intein systems. Indeed, in the last decade research on inteins has progressed in both industry and academia with a view towards commercialization.

While directed evolution and rational design principles have been used for obtaining better inteins for protein purification applications, the discovery of new and faster inteins in nature has also played a major role in the development of these technologies. The development of screening methods, versatile expression systems, modified purification resins and more convenient cloning systems in different patents over the years have occurred hand-in-hand with the evolution of better performing inteins. Although intein-based purification systems have come a long way, they have not yet reached their full potential of being a cheap, scalable, and universal purification platform for non-mAb proteins (both glycosylated and non-glycosylated). In particular, additional work needs to be done to make intein based purification systems truly indifferent to the protein being purified and to ensure rapid cleavage with minimal risk of product loss. The cost of intein-based purification methods might also be decreased through the use of non-chromatographic purification tags, which has already been demonstrated with elastin-like peptides and other examples (Hassouneh et al., 2010; Tian and Sun, 2011; Fan et al., 2018).

There exists immense opportunity to use inteins outside the protein purification space. Most of the opportunity lies in new therapies being developed to treat hitherto neglected diseases. For example, inteins have been used for protein *trans*-splicing in gene delivery applications (Li et al., 2008), adeno associated virus (AAV) capsid engineering (Muik et al., 2017) and for the development of Cas9 based gene therapies (Truong et al., 2015). Inteins have also been used for making pharmaceutical products in transgenic plants (Morassutti et al., 2002). Intein-based purification systems can potentially be used for the purification of AAV-based gene therapy products, and limited work has been done on the development of such systems. Further, intein based purification systems can also be used for the potential purification of extracellular vesicle (EV) based therapeutics, which are currently purified using affinity methods that are difficult to scale up (Konoshenko et al., 2018). Thus, it can be concluded that much work remains to be done to explore the full potential of inteins, but optimism is warranted for the current and upcoming advancements in intein technology.
